# Evening cortisol levels are prognostic for progression-free survival in a prospective pilot study of head and neck cancer patients

**DOI:** 10.3389/fonc.2024.1436996

**Published:** 2024-11-20

**Authors:** Elizabeth Cash, Isak Beck, Brooks Harbison, Christy Albert, Sandra E. Sephton

**Affiliations:** ^1^ Department of Otolaryngology–Head and Neck Surgery and Communicative Disorders, University of Louisville School of Medicine, Louisville, KY, United States; ^2^ University of Louisville Healthcare−Brown Cancer Center, Louisville, KY, United States; ^3^ Psychiatry and Behavioral Medicine, Medical College of Wisconsin, Madison, WI, United States; ^4^ Department of Psychology, Brigham Young University, Provo, UT, United States; ^5^ Department of Psychological and Brain Sciences, University of Louisville, Louisville, KY, United States

**Keywords:** head and neck cancer, cortisol, progression-free survival, interferon-gamma, circadian rhythm disruption

## Abstract

**Introduction:**

Cortisol rhythm disruptions predict early mortality in renal, colorectal, lung, and metastatic breast cancer. In head and neck cancer (HNC), various cortisol indices are known to correlate with adverse psychological and biological (e.g., inflammatory) outcomes, but links to mortality have yet to be demonstrated. We hypothesize that the prognostic value of diurnal cortisol aberrations will hold in HNC. Prior work leads us to predict that flattened or elevated diurnal cortisol profiles will be associated with elevations of serum inflammatory and tumor-promoting cytokines in this population, and that these immune markers would themselves predict poor progression-free survival.

**Method:**

We prospectively recruited a pilot sample of HNC patients (N=40) at a multidisciplinary HNC clinic. Most patients presented with late-stage oral/oropharyngeal cancer, were older than 50, male, and subsequently received combined-modality (surgery and/or radiotherapy with or without chemotherapy) treatment with curative intent. Saliva was collected twice daily for six days to assess diurnal slope, mean, waking, and evening cortisol levels. Serum was assayed for an exploratory panel of inflammatory and tumor-promoting cytokines. Two years post study-entry, disease progression and survivorship status were abstracted from medical records. Bivariate correlations, linear regressions, and Cox Proportional Hazards models tested hypotheses.

**Results:**

Elevations of evening cortisol and diurnal mean levels were each associated with shorter progression-free survival (evening: Hazard Ratio [HR]=1.848, 95% Confidence Interval [CI]=1.057-3.230, p=.031; diurnal mean: HR=2.662, 95% CI=1.115-6.355, p=.027). Bivariate correlations revealed that higher levels of the serum inflammatory marker interferon (IFN)-γ were linked with elevated evening (r=.405, p=.014) and mean (r=.459, p=.004) cortisol. Higher expression of IFN-γ also predicted poorer progression-free survival (HR=4.671, 95% CI=1.409-15.484, p=.012).

**Discussion:**

Elevated evening and diurnal mean cortisol were both prognostic; suggesting cortisol secretion is both dysregulated and elevated among patients who subsequently experienced accelerated disease progression. These exploratory data from 40 HNC patients mirror relationships between cortisol and survival identified among patients with numerous other tumor types. This pilot study highlights the need for research on effects of cortisol rhythm disruption among HNC patients. Future research in larger samples should also examine the role of inflammatory and tumor-promoting factors–both systemically and within the tumor microenvironment–as potential mediators of cortisol rhythm disruption.

## 1 Introduction

Among cancer patients, disrupted circadian rhythms are associated with poor quality of life and accelerated disease progression. Clinical and preclinical studies have linked circadian disruption to cancer initiation and progression (1–3). Paralleling this, poor central circadian control is linked with lower treatment efficacy in patients with cancer (2). Consequently, circadian rhythms are being increasingly considered in cancer research, with recent lines of inquiry examining circadian rhythms as a therapeutic target for new cancer treatments (4).

The hypothalamic-pituitary-adrenal (HPA) rhythm is an endocrine axis serving as the body’s principal circadian regulator through modulating the expression of the stress hormone cortisol. Typical HPA rhythm in healthy individuals is characterized by the expression of cortisol at high levels in the morning and nadir levels during sleep (5). Between 30-70% of advanced cancer patients show idiosyncratic changes in cortisol rhythm: unsynchronized peaks and troughs, erratic fluctuations, or consistently high or low levels (6). Viewed in aggregate, diurnal cortisol profiles of advanced cancer patients appear “flattened” (7, 8). Flattened cortisol rhythms are strongly linked with accelerated tumor growth (9–11) as well as early mortality from renal, lung, colorectal, and metastatic breast cancer (7, 8, 12, 13). Notably, elevation of evening cortisol levels - which contribute to flattened diurnal cortisol slopes - are prognostic for shorter survival among patients with ovarian cancer (14).

At the cellular level, cortisol coordinates the circadian rhythms of peripheral cells, including patterns of activity, growth and metabolism, mitosis, DNA repair, apoptosis, senescence, and autophagy. When circadian rhythms are disrupted, changes in these processes can lead to the acquisition of cancer hallmarks by tumor cells and result in rapid proliferation and metastasis, escape from apoptosis, angiogenesis, drug resistance, and cause an immunosuppressive shift in the tumor microenvironment that favors tumor cell proliferation (1, 15–18). Additionally, tumors with the ability to upregulate their own internal circadian clocks grow faster, leading to shorter host survival (19).

Scholars have recently begun to examine relationships between cortisol and oncologic processes among head and neck cancer (HNC) patients. Single time point salivary measures have demonstrated feasibility, including among patients with cancers in the oral cavity and those suffering from xerostomia (20). Studies have revealed that HNC-related elevations in basal cortisol can persist over time (21), can become increasingly pronounced with advancing disease (22, 23), and may be associated with increased incidence of regional head and neck metastases (24). Among newly diagnosed HNC patients, elevated diurnal mean and evening salivary cortisol, measured on a single day, have also been associated with poorer HNC-specific health-related quality of life (25). However, no studies to our knowledge have rigorously assessed diurnal cortisol aberrations among patients with HNC, which requires multiple samples over successive days of data collection (26).

Among HNC patients, aberrations in cortisol indices are known to correlate with adverse psychological and biological outcomes. This may be due in part to the direct effects of cortisol on immune function: Cortisol is an important driver of immune cell trafficking and cytotoxic activity (27–29). In an epidemiological study of HNC patients, diurnal salivary cortisol aberrations–as well as anxiety, depression, poor sleep quality, fatigue, and reduced quality of life–were linked to elevations in the serum inflammatory markers C-reactive protein (CRP) and IL-6 (30). In a case-control study examining a broad array of cytokines, only four analytes differed significantly between HNC patients and controls: patients had lower serum interferon (IFN)-γ, interleukin (IL)-13, macrophage inflammatory protein-1β (MIP-1β); and elevated levels of IFN-γ-inducible protein-10 (IP-10; 31). In contrast, another study that compared data from HNC patients with controls (32) found a different pattern of results, with significantly elevated IFN-γ. IL-13 was not measured, but similar to the study by Kaskas et al. (31), IP-10 was elevated, as were additional factors including IL-1ra, IL-2, IL-5, IL-6, IL-8, and IL-17. Links between diurnal cortisol, cytokines, and HNC mortality have yet to be demonstrated.

In this prospective pilot sample of newly diagnosed HNC patients, we aimed to rigorously assess diurnal cortisol rhythms, using methods appropriate for reliable calculation of diurnal slope, waking, evening, and diurnal mean cortisol values (26, 33). We hypothesized that disruptions in diurnal cortisol rhythm collected during the diagnostic and treatment planning phase would be associated with poorer subsequent two-year progression-free survival. In secondary, exploratory analyses, we tested associations of diurnal cortisol variables with serum inflammatory and tumor-promoting cytokines; and where appropriate, we tested the prognostic value of these cytokines.

## 2 Methods

### 2.1 Participants

Participants in this study were prospectively recruited as part of a pilot study on circadian rhythm disruption among patients with primary HNC. After IRB approval (study #14.0607), patients who were recently diagnosed with new primary HNC were invited to participate during their initial treatment planning appointment at a Multidisciplinary Head and Neck Cancer Clinic. Those who were not fluent in English, did not have pathologically-proven malignancy, would be treated at another facility, had comorbid alcohol abuse, had a history of severe psychiatric illness, lived farther than 120 miles from the treatment center, or were severely immunocompromised (e.g., HIV+; concurrent use of immunosuppressants for organ transplant) were excluded.

### 2.2 Procedures

Data collection occurred between September 2014 and June 2016. A total of 197 patients were screened; of those, 132 met inclusion criteria. During the initial treatment-planning appointment, eligible participants were approached by study personnel and invited to participate. Reasons for declining included: acute pain or distress (n=6); limited time to complete data collection prior to beginning treatment (n=16); and lack of interest in participation (n=55).

A total of 55 participants enrolled. A blood draw was collected at the time of enrollment for assessment of serum inflammatory and tumor-promoting cytokines. Participants were instructed on how to collect and store saliva samples at waking and bedtime for the subsequent six days. They were also instructed on the use of actigraphy watches, from which data were used to confirm participant adherence to the saliva sample timing instructions. Upon return of data, participants were compensated with a $50 prepaid gift card. Fifteen of 55 patients did not provide complete data; thus, the final sample included 40 participants.

### 2.3 Measures

#### 2.3.1 Sociocultural and demographic variables

Participants self-reported on demographic characteristics, including age, sex, race, marital status, educational achievement, ethnicity, and annual income.

#### 2.3.2 Clinical variables

Information about participant disease status and treatment was abstracted from medical records, including pack-years of smoking history, cancer stage at diagnosis, cancer site, human papillomavirus (HPV) status, current medications, and subsequent cancer treatment received. Two years post study-entry, disease progression and survivorship status were abstracted from medical records, yielding two variables for use in Cox regressions: tracking time (number of days from study entry to the date of recorded progression of disease or death; or for those with no disease progression or death, tracking stopped at two years/730 days), and status (a dichotomous variable indicating whether or not subjects experienced disease progression or death). The two variables, tracking time and status, were used in survival analyses to indicate progression-free survival.

#### 2.3.3 Cortisol

##### 2.3.3.1 Data cleaning and scoring procedures for diurnal salivary cortisol

Cortisol was collected via salivary sampling at waking and bedtime on six consecutive days. Cotton Salivettes (Sarstedt, Inc.; Newton, NC) were used, and dates and times were recorded by participants at each sample collection. Participants were asked to refrain from eating, drinking, smoking, brushing their teeth, using mouthwash, and chewing gum for half an hour before each sample collection. They were instructed to hold the cotton in their mouths for around two minutes or until saturated to ensure sufficient saliva was collected. Participant adherence to the saliva collection schedule was monitored via actigraphy data (see Salivary Data Collection Procedure Adherence, below). Participants stored completed saliva samples in their refrigerator, typically for one to two days, before returning them to the research team. Upon receipt at the lab, a trained research assistant centrifuged, aliquoted, and stored saliva samples at -80°C.

Assays were conducted using an enzyme-linked immunoassay (ELISA) developed specifically for saliva (Salimetrics, Inc., State College, PA). The lower limit of assay sensitivity was .003 µg/dL. The cortisol inter-assay CV was 5.9% for low controls and 5.9% for high controls; intra-assay CVs were 5.8% for low controls and 2.9% for high controls.

##### 2.3.3.2 Salivary data collection procedure adherence

As participant compliance with salivary data collection procedures/schedule is critical (34), actigraphy data were used to verify salivary data collection procedural compliance. Participants wore an actigraphy watch (Mini-Motionlogger; Ambulatory Monitoring Systems, Inc., Ardsley, N.Y.) during the six days of salivary data collection. Watches were removed in situations where a watch was not allowed to be worn (e.g., the watch would interfere with medical equipment). There were a few instances wherein the watches were briefly removed (e.g., during a clinical procedure), and minutes of data from those instances were deleted. These instances were few and short in span (e.g., <30 minutes) and did not overlap with saliva collection times.

##### 2.3.3.3 Data cleaning and transformation for cortisol variables

Of the entire array of saliva samples (N=552), n=79 samples were modified: For 6.3% (n=35) of bedtime samples, collection time was modified based on accelerometry data (e.g., if actigraphy showed the patient was sleeping at the minute of self-reported saliva collection; typically the difference was <5 minutes). When self-reported waking saliva collection times differed from the accelerometry-based estimate of first awakening by <15 minutes, accelerometry wake times were favored due to their objectivity; this occurred for 7.9% (n=44) of the wake samples. Finally, 6.7% (n=37) were omitted from analyses based on *a priori* exclusion criteria: values greater than 4 SDs from the mean (n=4) or waking values with large irreconcilable discrepancies between self-reported collection time and accelerometry data (e.g., >15 minutes; n=33). These steps yielded 515 samples for analysis. The average wake sample collection time was 07:57 (SD=2:11), and average bedtime collection time was 23:40 (SD=2:51).

Because cortisol data are typically skewed due to natural circadian-linked elevations in the morning (35), raw salivary cortisol values were log-transformed to meet parametric analytic assumptions. Calculated cortisol variables included diurnal cortisol slope, log mean waking value, log mean bedtime value, and diurnal log mean cortisol. The diurnal cortisol slope was calculated as the unstandardized β of log-transformed cortisol regressed on collection time over all 12 samples. Mean wake values were calculated using all 6 log-transformed wake samples; mean evening values were calculated using all 6 log-transformed bedtime samples. Diurnal cortisol mean was calculated using all 12 log-transformed waking and bedtime samples (26).

#### 2.3.4 Serum inflammatory and tumor-promoting cytokines

A trained phlebotomist drew single blood samples using Vacutainer (Beckton, Dickinson and Company; Franklin, NJ) tubes. Serum was collected as close to study entry as possible, and always prior to definitive chemoradiation. Since biomarkers of interest are known to exhibit circadian patterns of release in systemic circulation (36), the timing of blood draw was restricted as much as possible. Mean blood collection time was 11:38 a.m. (SD=30 min; median=11:41 a.m.); none were postprandial. Serum aliquots were frozen at -80°C within 1 hour of blood draw. A MILLIPLEX MAP Human High Sensitivity T Cell Magnetic Bead Panel immunoassay (MilliporeSigma; Billerica, MA) was used to quantify granulocyte-macrophage colony-stimulating factor (GM-CSF), IFN-γ, IL-1β, IL-5, IL-6, IL-7, IL-10, IL-12, IL-13, IL-17α, macrophage inflammatory protein (MIP)-1α, MIP-1β, and tumor necrosis factor (TNF)-α. Assay plates were imaged on a Luminex 100/200, and data were analyzed using Luminex xPONENT 3.1 software (ThermoFisher Scientific; Waltham, MA). Respectively, the detection limits, intra-assay, and inter-assay CVs were: GM-CSF (1.22, 9.38%, 5.63%), IFN-γ (0.61, 13.82%, 5.18%), IL-1β (0.49, 6.93%, 1.52%), IL-5 (0.49, 3.95%, 5.58%), IL-6 (0.18, 6.41%, 1.82%), IL-7 (0.37, 1.36%, 4.25%), IL-10 (0.73, 2.37%, 2.58%), IL12 (0.49, 2.10%, 3.96%), IL-13 (0.24, 12.71%, 4.48%), IL-17α (0.73, 7.61%, 4.57%), MIP-1α (0.31, 1.73%, 2.72%), MIP-1β (0.92, 4.91%, 5.46%), and TNF-α (0.43, 10.56%, 6.7%).

### 2.4 Analyses

#### 2.4.1 Primary analysis

Cox proportional hazards models were used to test the hypothesis that disrupted diurnal cortisol rhythms would be associated with poorer two-year progression-free survival.

#### 2.4.2 Secondary analysis

Our *a priori* hypothesis guiding secondary, exploratory analyses was that serum inflammatory and tumor-promoting cytokines would be associated both with cortisol variables and survival outcomes. Bivariate correlations tested associations between cortisol variables, survival variables, and serum inflammatory and tumor-promoting cytokines. Cytokines that were significantly correlated both with any cortisol variable and either survival variable were further explored in secondary analyses using linear regressions, in which cortisol variables were predictors and cytokines were outcome variables. For cytokines that demonstrated a significant association with cortisol in regression analyses, Cox models incorporating both tracking time and status were employed to test the prognostic value of the cytokine for two-year progression-free survival. All statistical tests were 2-sided with alpha set at .05 (SPSS 29; IBM, Armonk, New York). Because these were exploratory analyses, we did not correct for multiple comparisons (37).

Spearman rank correlations were used to assess the contributions of traditional prognostic indicators, including cancer stage, site of disease, age at diagnosis, sex, race, marital status, and tobacco history in pack-years. Those that correlated with both the predictor for each model (e.g., cortisol or IFN-γ) and survival, were considered possible confounds.

## 3 Results

Demographic and clinical characteristics of the pilot sample are provided in **Table 1**. The sample was predominantly married White males, who had attended some high school through some college (e.g., vocational degree), and earned less than $70,000 per year. The average number of days between initial cancer diagnosis and enrollment was 7.7 (SD=17.22, range 0-87). Because of changes in practice patterns at our clinic during the time of study recruitment, 12 patients had undergone an extirpative surgical procedure prior to collection of salivary and serum data. However, statistical comparison of summary statistics for each study variable (salivary cortisol indices and serum inflammatory/tumor-promoting cytokines) revealed no significant differences between preoperative and postoperative patients (all p’s >.099); data from all pre- and post-operative patients were included in analysis.

**Table 1:** Sample characteristics (N=40).

		N	%	Median	M	SD
Age at Diagnosis (range 24 - 73)		40		57.0	56.7	10.9
Pack-Years (range 0 - 80)		40		2.0	16.8	22.5
Male Sex		24	60.0%			
Marital Status	Never married	5	12.5%			
	Married	23	57.5%			
	Divorced	7	17.5%			
	Widowed	3	7.5%			
	Separated	1	2.5%			
Race	White	33	82.5%			
	Non-White	6	15.0%			
Site of Disease	Oral	11	27.5%			
	Oropharyngeal	11	27.5%			
	HPV-positive	9	81.8%			
	HPV-negative	2	18.2%			
	Laryngeal	6	15.0%			
	Other	10	25.0%			
Stage	Early	12	30.8%			
	Late	29	69.2%			
Treatment Received	Surgery Only	6	15.0%			
	Surgery + Radiation	8	20.0%			
	Surgery + Radiation + Chemotherapy	12	30.0%			
	Radiation +/- Chemotherapy	14	35.0%			
Diurnal Cortisol	Diurnal Slope (log(μg/dL/hr))	39		-.092	-.085	.057
	Waking Cortisol (μg/dL)	40		.391	.431	.243
	Evening Cortisol (μg/dL)	39		.092	.178	.265
	Diurnal Mean (μg/dL)	40		.241	.303	.225
Events at Two-Year Follow-Up	Locoregional Recurrence	4	10.0%			
	Distant Metastases	5	12.5%			
	Deaths	2	5.0%			
Two-Year Progression Free Survival, Months (range 0 - 24)		40		6.7	12.2	8.3

*HPV, human papillomavirus.

### 3.1 Tests of hypotheses

#### 3.1.1 Primary analyses

**Figure 1:**
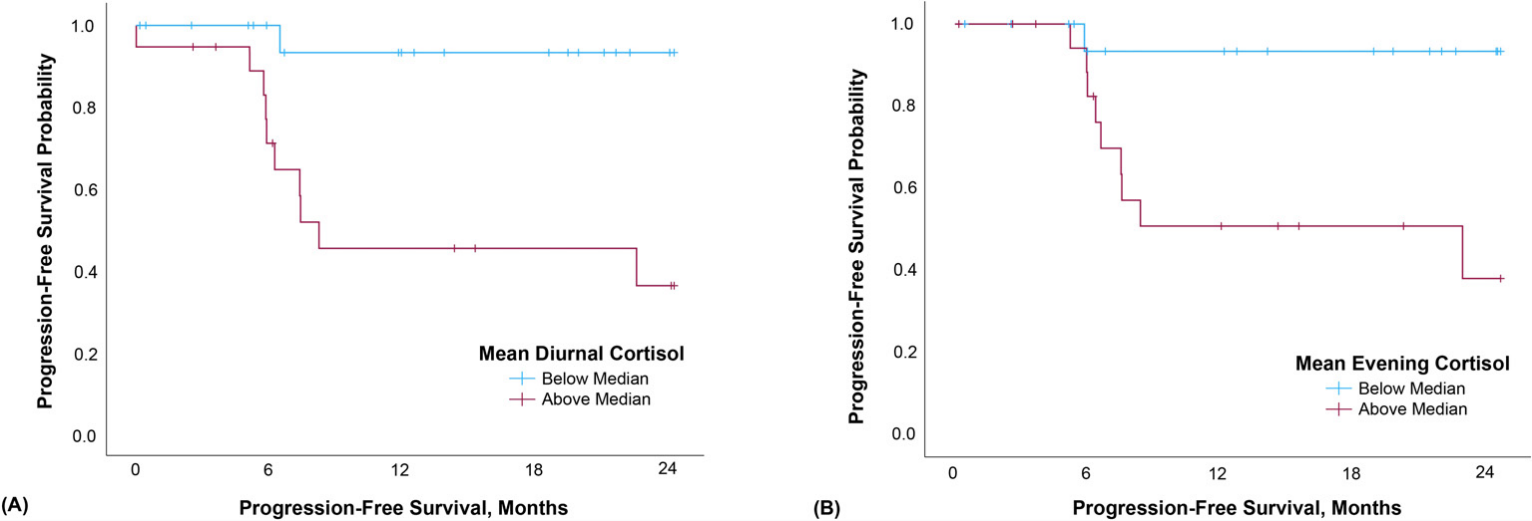
Mean diurnal **(A)** and evening **(B)** cortisol were both significantly associated with progression-free survival. Data were split at the median and Kaplan-Meier curves generated for illustrative, not analytic, purposes.

The proportional hazards model was constructed with diurnal cortisol slope entered as a continuous variable; however, in this small sample, the slope values did not meet assumption of linearity, so the Cox model was reconstructed entering diurnal slope stratified at the median value: below median/steeper slopes N=21, M=-.128, SD=.028; above median/flattened slopes, N=19, M=-.039, SD=.042. Diurnal slope was not significantly associated with progression-free survival (HR=3.495, 95% CI=0.901-13.556, p=.070). Elevated evening cortisol and diurnal cortisol mean were both significantly associated with poorer progression-free survival (evening: HR=1.848, 95% CI=1.057-3.230, p=.031; diurnal mean: HR=2.662, 95% CI=1.115-6.355, p=.027; **Figure 1**).

#### 3.1.2 Secondary exploratory analyses

Because recent data from our laboratory have highlighted inflammation as a mediator of survival effects in HNC (38), we explored the role of inflammatory and tumor-promoting immune markers in the cortisol–survival analyses. Immune markers included in the analyses are listed in **Table 2**.

**Table 2:** Raw mean and SD values for an exploratory panel of serum inflammatory and tumor-promoting immune markers and their associations with cortisol and survival parameters.

Analyte (pg/mL)	Mean	SD	Evening Cortisol	Mean Diurnal Cortisol	Progression-Free Survival Status
GM-CSF	54.028	45.404	.189	.254	.193
IFN-γ	11.801	7.192	.335^*^	.435^**^	.427^**^
IL-1β	1.696	1.021	.407^*^	.373^*^	.211
IL-5	3.929	5.644	.236	.230	.433^**^
IL-6	5.015	3.629	.187	.224	.410^*^
IL-7	12.456	5.296	.322	.323^*^	.307
IL-10	9.180	7.345	.315	.376^*^	.254
IL-12	3.414	3.456	.110	.082	-.066
IL-13	36.364	52.989	.098	.122	.088
IL-17α	15.050	13.315	.393^*^	.408^*^	.277
MIP-1α	33.152	14.466	.278	.233	.394^*^
MIP-1β	31.812	11.820	.199	.159	.262
TNF-α	7.629	3.405	.093	.176	.275

Bivariate Spearman correlations between log transformed immune markers, diurnal cortisol measures, and progression-free survival were performed. IFN-γ was the only marker significantly correlated with both diurnal cortisol and progression-free survival and therefore the only marker further explored in secondary analyses.

*p <.05.

**p <.01.

Bivariate Spearman correlations revealed no significant association of any cytokine with diurnal cortisol slope, waking cortisol, or progression-free survival tracking time. However, one of the 13 cytokines studied, IFN-γ, was associated both with cortisol (evening and mean levels) and progression-free survival (status; **Table 2**), suggesting it may meet criteria for statistical mediation in a larger sample of patients. On this pilot basis, linear regressions revealed higher IFN-γ levels were significantly associated with evening cortisol (r=.405, p=.014) and diurnal mean cortisol (r=.459, p=.004), but not diurnal cortisol slope. Thus, an exploratory Cox regression was performed with IFN-γ entered as a continuous variable. Higher IFN-γ predicted poorer progression-free survival (HR=4.671, 95% CI=1.409-15.484, p=.012).

In assessing for possible confounds, we observed that no clinical or demographic variables were associated with both the predictor and progression-free survival. Thus, no adjustments were made to tests of hypotheses.

## 4 Discussion

### 4.1 Diurnal cortisol predicts progression-free survival

Our study provides the first exploratory evidence suggesting that elevated evening and diurnal mean cortisol are prognostically relevant in HNC. These data suggest future studies should test hypotheses that cortisol secretion is both dysregulated and elevated among HNC patients who subsequently experience accelerated disease progression. The current finding is consistent with prior work demonstrating strong links between flattened cortisol rhythms and early mortality among renal, lung, colorectal, and metastatic breast cancer patients (7, 8, 12, 13). Further, results underscore the notion that evening cortisol may represent a key physiological marker of cancer-relevant circadian disruption among patients with HNC, and that evening levels may be an adequate standalone measure of HPA rhythm disruption relevant to survival.

**Figure 2:**
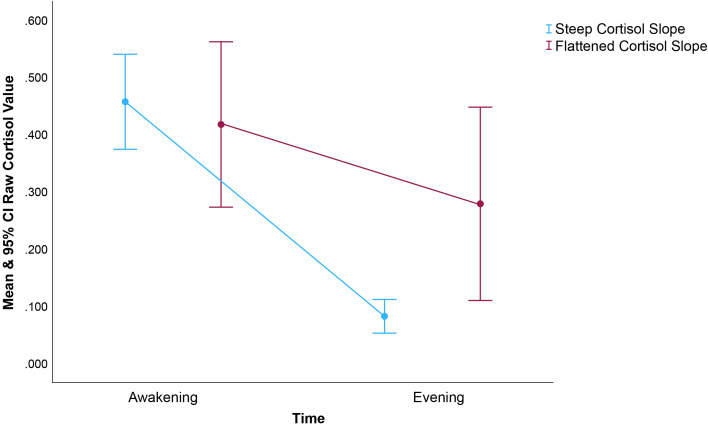
Raw mean and 95% CI cortisol based on a median split of the diurnal cortisol slope within our sample. As noted, prior studies found diurnal cortisol slope prognostic for cancer mortality, a finding not replicated in this small (n=40) sample of HNC patients. Rather, we found that only the evening and overall mean cortisol levels predicted survival. To inform future research, we felt it important to describe interrelationships between cortisol summary variables. It’s worth noting that evening cortisol appears to distinguish steep from flattened slopes more distinctly than do waking values. The restricted CI for evening cortisol among those with steep, healthier cortisol is also interesting. This figure demonstrates the magnitude of the difference in evening cortisol level associated with differences in cortisol slope.

Diurnal HPA rhythms are typically marked by high cortisol levels in the morning hours (peaking 30-45 minutes after first waking) with steady declines in expression level throughout the day until a nadir is reached during sleep. Evening samples may, on their own, be sufficient to discriminate among patients with healthy ‘steep’ versus unhealthy ‘flattened’ cortisol rhythms (39). Elevated evening cortisol levels have been shown to predict early cancer mortality among ovarian cancer patients (14). Consistent with this, our pilot data demonstrate that evening cortisol predicts poorer progression-free survival. **Figure 2** suggests that at an aggregate level among this small sample of HNC patients, evening cortisol may distinguish steep from flattened cortisol slopes more effectively than waking values. As often noted, variability appears lower in evening cortisol levels in comparison with waking, represented in our figure by the restricted confidence interval for evening cortisol. Had our sample been larger, we may have also observed prognostic effects of diurnal cortisol slope. Further, as different clinical conditions are associated with markedly different profiles of circadian cortisol secretion aberrations, we suggest that future research not exclude waking cortisol levels and slopes. Future, larger studies should also explore the prognostic value and psychophysiological relevance of intraindividual variability in cortisol rhythms, including for example day-to-day slope stability (40).

Our time-consistent six-day schema for cortisol sampling represents a major strength of this study. Robust measurement of chronic physiological stress via diurnal cortisol sampling at consistent points across multiple days (26) grants us heightened rigor and more consistent results. Most studies examining cortisol among HNC patients employ simpler data collection strategies, such as single-day or single time-point cortisol measures (21, 22, 24, 30). Variability in results across studies is compounded by differing time points at which samples are collected and variance in calculation methodologies for cortisol summary variables, making summative conclusions difficult to draw. Moreover, we address a shortcoming of prior cancer research by examining these relationships over the course of multiple days in the short timeframe between cancer diagnosis and treatment planning, a period relatively free of the potential confounds of medically-induced treatment effects.

Among cancer patients, multiple factors may contribute to disrupted HPA rhythms. Diagnosis and treatment may exact psychological stress that is frequent and repeated, conveying a high risk for depression and possibly leading to HPA axis dysregulation (41, 42). Among presurgical oral cavity cancer patients, those presenting with greater symptoms of depression exhibit higher circulating levels of cortisol—measured at a single time point—compared to those with benign disease (23). In turn, elevated depressive symptoms predict disease progression and poorer treatment outcomes, including greater neck node metastases (43), poorer tumor response to medical treatment, and poorer overall survival in HNC (38, 44, 45). Depression and concomitant inflammation can exacerbate HPA dysregulation (46). Thus depression and cortisol dysregulation may feed on one another, creating a cycle that negatively influences cancer treatment and survivorship outcomes (46, 47).

Sleep disruption is another likely component in the nexus of cancer-related depression, cortisol rhythm disruption, and inflammation (48, 49). Animal studies show that both acute and chronic stress profoundly affect sleep architecture and circadian rhythms (50). Sleep loss delays the recovery of the HPA axis from stimulation, possibly involving an alteration in negative glucocorticoid feedback regulation (51). Finally, tumors are known to disrupt multiple physiological processes including neural, endocrine, metabolic, and immune functions (9). Each of these may impact sleep – comprising multiple potential indirect pathways of cancer effects on sleep and circadian regulation (52, 53).

Preclinical *in vivo* models of cancer have similarly demonstrated that psychosocial factors affect tumor cells, modulate anti-cancer immunity, and impact the tumor microenvironment; providing insights for potential interventions aimed at slowing cancer progression and improving treatment response (54). Among HNC patients, burgeoning evidence points to psychoneuroimmune relationships across various immune markers and tumor-growth factors. In an epidemiological study of HNC patients, IL-6 was observed to be a key marker linked with diurnal salivary cortisol aberrations and anxiety, depression, poor sleep quality, fatigue, and reduced quality of life (30). Among patients with non-HPV associated HNCs, higher reported levels of perceived stress, anxiety and depressive symptoms have been linked to circulating levels of the tumor angiogenesis marker vascular endothelial growth factor (VEGF; 55). Similar patterns have been shown in studies assessing the effects of cortisol exposure, showing that it regulates circadian rhythms of activity, growth and metabolism in peripheral cells; and drives immune cell trafficking and cytotoxic activity (27–29). Given the similarities observed in psychoneuro-endocrine and -immune relationships across multiple cancer types, these pathways warrant further study among HNC patients.

### 4.2 IFN-γ predicts progression-free survival

These exploratory analyses revealed IFN-γ was associated with progression-free survival in this small sample of newly-diagnosed HNC patients. We view these interesting results with caution for a number of reasons. First, IFN-γ displays both tumor-promoting and tumor-suppressive roles in oncologic processes. This cytokine—produced predominantly by natural killer and cytotoxic T cells—promotes tumor defenses by activating type 1 (cellular) immunity (56) and suppressing type 2 (global allergy and inflammatory) responses (57). As such, IFN-γ has long been considered central in antitumor immunity (58). It is known to enhance immune responses against tumors by upregulating tumor expression of MHC class 1 molecules, making cancer cells easier for immune cells to recognize and destroy (59). IFN-γ also enhances the cytotoxic activity of NK cells and cytotoxic T lymphocytes (60). Indeed, IFN-γ has been used as an adjuvant treatment based on its cytostatic, pro-apoptotic, antiproliferative, and anti-angiogenic functions (58).

Paradoxically, IFN-γ also has a role in tumor promotion: cancer cells exposed to IFN-γ demonstrate increased capability for immune evasion (60). Within the tumor microenvironment (TME), T cell production of IFN-γ creates a widespread cytokine field shared by most tumor cells as well as infiltrating immune cells. Thus, effects of IFN-γ are likely active within a broader tumor-associated cytokine field, rather than by very discrete cytokine hotspots (61). However, concentrations of IFN-γ in the TME determine whether the function of this cytokine is pro- or anti-tumorigenic (58). IFN-γ contributes to tumor promotion or eradication both directly and indirectly by cooperating with other TME mediators, so effects of this cytokine can likely not be understood without considering other aspects of the intracellular milieu (58). With regard to effects on tumor growth, levels of IFN-γ within the TME are probably more informative than are the serum levels measured here. However, serum measurement may still point to valid processes, as changes in serum cytokines may mirror those in the TME in some instances (62, 63). Research designs contrasting systemic biomarker expression against potential cytokine hotspots in the TME will be highly informative.

Among HNC patients, recent research also highlights contrasting findings when assessing IFN-γ: In one study, downregulated serum IFN-γ was noted in patients compared with controls (31). In another, elevated serum levels were observed among node-negative patients who demonstrated better disease-specific survival (32). In light of our observation that elevated IFN-γ associates with worse HNC outcomes, these findings underscore the need for greater clarity into the roles of IFN-γ in HNC.

### 4.3 Implications

Our results point to additional targets for future examination in larger studies, including circadian rhythm disruption that may extend to rest-activity rhythms and sleep parameters. A recent examination highlighted measurement strategies for estimating circadian disruption, and found that both rest-activity and HPA rhythms were consistently prognostic for cancer mortality (64). In support, our group has previously shown rest-activity rhythms to be prognostic in HNC (44). Disruptions at the cellular level should also be given consideration. For example, telomerase is regulated by the circadian time-keeping machinery, coordinated in peripheral cells by cortisol rhythms (65). In turn, telomerase is a crucial regulator of cancer progression that induces replicative immortality and inhibits apoptosis. As such, it should be a target in future studies of circadian effects on tumor progression (66). To our knowledge, none of these associations have been studied in HNC patients.

Clinically, immunotherapy is becoming increasingly utilized for HNC. Circadian disruption may have deleterious effects on immunotherapy outcomes: HNC patients who receive infusion treatments later in the day (versus early morning when the adaptive immune response may be under better circadian regulation) may suffer suboptimal survival outcomes (67). Potentially compounding the effects of treatment timing misalignment, cortisol dysregulation has the potential to influence immunotherapy efficacy. Rises in circulating glucocorticoids induced by chronic stress lead to a reduced ability to mount effective anti-programmed death (PD)-1-induced anti-tumor immune responses in mice, and similar effects were observed after corticosteroid administration (68). Patterns parallel to this have been seen among corollary patient samples: higher distress has been associated with elevated circulating glucocorticoids which, in turn, have been associated with suboptimal anti-PD-1 therapeutic response (68), shorter progression-free and overall survival times (69), and altered IFN-γ signaling pathways (70). As these stress-related immune marker and cancer effects have been implicated in immunotherapy outcomes (71), these intriguing findings warrant further, prospective study where enhanced predictive capacity could potentially be furnished through assessment of diurnal fluctuations of endogenous glucocorticoid (i.e., salivary cortisol) expression. Studies of this nature would also allow for better elucidation of the potential for mediation of these effects by IFN-γ.

Our results point to clinically-relevant targets with the potential to improve cancer patient well-being. For example, development of a clinically-available, robust diurnal salivary collection paradigm could provide valuable information to oncologists, as these data might be useful for flagging cases at elevated risk for accelerated disease progression. Similarly, depression stands out as a highly feasible target for renewed clinical attention (38). Behavioral interventions, including cognitive-behavioral therapy (CBT) and Mindfulness-Based Stress Reduction, have shown promise in reducing depression and managing cortisol dysregulation among patients with other cancer types (72–74). Similarly, screening for poor sleep quality among HNC patients using validated sleep evaluation tools - such as actigraphy - can help identify those at risk of persistent poor sleep quality and allow for early, effective interventions (e.g., CBT) targeting sleep disturbances and related symptoms (75, 76), which may in turn offer benefit for managing cortisol dysregulation (74).

### 4.4 Limitations

This study is limited by the small sample size, small number of subjects who experienced cancer progression and death, and large number of serum inflammatory and tumor-promoting cytokines analyzed. Our methodology did not allow us to comment on whether variations in serum cytokine measurements were a result of host versus tumor effects, nor can we comment on aspects of the tumor microenvironment that may have played a role in tumor response and treatment outcomes. These limitations make it difficult to draw firm conclusions from our data. Instead, our pilot study suggests that rigorous, multi-day diurnal salivary data collection methods are feasible even among HNC patients where potentially high levels of pain and interference from the tumor location have often been considered impediments to data collection.

### 4.5 Conclusions

This pilot study suggests for the first time that diurnal cortisol expression has prognostic relevance in HNC. We observed that elevated evening and diurnal mean cortisol were both prognostic, suggesting cortisol secretion is both dysregulated and elevated among patients who subsequently experienced accelerated disease progression. Our data represent a valid approach to robustly measuring diurnal cortisol expression among patients with HNC, and add to a growing body of literature suggesting diurnal cortisol aberrations portend poorer cancer outcomes. Future research in larger samples could add more definitive characterization to these findings, elucidate the role of psychosocial factors at this nexus, and further examine the role of inflammatory and immune markers (both systemically and within the tumor microenvironment) as potential mediators of the tumor-promoting effects of cortisol rhythm disruption.

## Data Availability

The raw data supporting the conclusions of this article will be made available by the authors, without undue reservation.
